# Highly efficient conversion of mouse fibroblasts into functional hepatic cells under chemical induction

**DOI:** 10.1093/jmcb/mjad071

**Published:** 2023-11-23

**Authors:** Zhi Zhong, Jiangchuan Du, Xiangjie Zhu, Lingting Guan, Yanyu Hu, Peilin Zhang, Hongyang Wang

**Affiliations:** Fudan University Shanghai Cancer Center, Department of Oncology, Shanghai Medical College, Fudan University, Shanghai 200032, China; National Center for Liver Cancer, Naval Medical University, Shanghai 201805, China; National Center for Liver Cancer, Naval Medical University, Shanghai 201805, China; National Center for Liver Cancer, Naval Medical University, Shanghai 201805, China; Institute of Metabolism and Integrative Biology, Fudan University, Shanghai 200438, China; National Center for Liver Cancer, Naval Medical University, Shanghai 201805, China; National Center for Liver Cancer, Naval Medical University, Shanghai 201805, China; National Center for Liver Cancer, Naval Medical University, Shanghai 201805, China; Fudan University Shanghai Cancer Center, Department of Oncology, Shanghai Medical College, Fudan University, Shanghai 200032, China; National Center for Liver Cancer, Naval Medical University, Shanghai 201805, China

**Keywords:** direct cell reprogramming, chemicals, fibroblasts, hepatocyte-like cells, high efficiency, regenerative medicine

## Abstract

Previous studies have shown that hepatocyte-like cells can be generated from fibroblasts using either lineage-specific transcription factors or chemical induction methods. However, these methods have their own deficiencies that restrict the therapeutic applications of such induced hepatocytes. In this study, we present a transgene-free, highly efficient chemical-induced direct reprogramming approach to generate hepatocyte-like cells from mouse embryonic fibroblasts (MEFs). Using a small molecule cocktail (SMC) as an inducer, MEFs can be directly reprogrammed into hepatocyte-like cells, bypassing the intermediate stages of pluripotent and immature hepatoblasts. These chemical-induced hepatocyte-like cells (ciHeps) closely resemble mature primary hepatocytes in terms of morphology, biological behavior, gene expression patterns, marker expression levels, and hepatic functions. Furthermore, transplanted ciHeps can integrate into the liver, promote liver regeneration, and improve survival rates in mice with acute liver damage. ciHeps can also ameliorate liver fibrosis caused by chronic injuries and enhance liver function. Notably, ciHeps exhibit no tumorigenic potential either *in vitro* or *in vivo*. Mechanistically, SMC-induced mesenchymal-to-epithelial transition and suppression of SNAI1 contribute to the fate conversion of fibroblasts into ciHeps. These results indicate that this transgene-free, chemical-induced direct reprogramming technique has the potential to serve as a valuable means of producing alternative hepatocytes for both research and therapeutic purposes. Additionally, this method also sheds light on the direct reprogramming of other cell types under chemical induction.

## Introduction

The liver is a pivotal organ in the human body. By regulating important physiological processes, such as protein production, lipid metabolism, and xenobiotic detoxification, the liver helps to maintain body homeostasis (Hengstler et al., 2005). Liver function impairment caused by various diseases can significantly harm human health and even lead to patient death (Xiao et al., 2019). Liver transplantation is often the only curative treatment for end-stage liver diseases (Marroni et al., 2018). Additionally, hepatocyte transplantation can serve as an alternative therapeutic approach, especially for hereditary metabolic liver diseases (Dhawan et al., 2010). However, the shortage of organs and insufficient supply of primary hepatocytes are creating a large unmet medical need. Scaled generation of mature functional hepatocytes *in vitro* might be the best solution to this problem. These functional human hepatocytes can be used not only for therapeutic purposes but also for disease modeling, drug toxicity evaluation, and bio-artificial liver construction (Gomez-Lechon et al., 2004; Castell et al., 2006; Carpentier et al., 2009; Cherry and Daley, 2012).

Previous studies have shown that fibroblasts can be directly converted into hepatocyte-like cells through the ectopic expression of a set of specific transcription factors (Huang et al., 2011; Sekiya and Suzuki, 2011; Du et al., 2014; Huang et al., 2014). However, the method of genetic manipulation has certain deficiencies, including low efficiency, a long and complex gene transduction process, and potential safety risks. These flaws greatly hamper the clinical usability of transcription factor-induced hepatocyte-like cells (iHeps). To solve these problems, researchers have made many attempts to optimize reprogramming protocols. For instance, it was later discovered that mouse fibroblasts could be converted into iHeps by a single transcription factor (FOXA1, FOXA2, or FOXA3) in combination with several small molecules, including CHIR99021, RepSox, VPA, Parnate, TTNPB, and Dznep (Guo et al., 2017), which increased the induction efficiency while reducing the use of exogenous genes. With further investigation, a recent report showed that mouse embryonic fibroblasts (MEFs) could also be converted into hepatocyte-like cells by using only chemicals (Bai et al., 2023). However, depending on the chemical used, this protocol only induced a conversion of 1%–2%, with a maximum of 15%, of MEFs into hepatocyte-like cells over a 35-day period. More importantly, the reprogrammed hepatocyte-like cells were Ki67-positive cells with proliferation ability *in vitro*, which resembled the characteristics of hepatoblasts (immature hepatic progenitor cells), rather than those of mature hepatocytes. The low reprogramming efficiency, long induction period, and immature features of the reprogrammed cells suggest that this method may not be the optimal approach for generating hepatocyte-like cells that are suitable for clinical use.

Here, we report that a small molecule cocktail (SMC) containing SB431542, CHIR99021, BIX01294, LDN193189, and DAPT can effectively convert both MEFs and mouse adult fibroblasts (MAFs) into hepatocyte-like cells within a 12-day induction process. These chemical-induced hepatocyte-like cells (ciHeps) exhibit hepatic characteristics and functions, and the conversion efficiency of this method can reach up to 80%, which is significantly higher than those of previous protocols. Transplanted ciHeps generated through this method can successfully integrate into the liver and express hepatocyte markers, thus promoting liver regeneration and markedly increasing mouse survival rates following acute liver failure (ACLF). Furthermore, these ciHeps can effectively reduce liver fibrosis and improve liver function. Notably, MEF-derived ciHeps have been demonstrated to be a safe cell source, as they exhibit no tumorigenic potential either *in vitro* or *in vivo*. Mechanistically, SMC-induced mesenchymal-to-epithelial transition (MET) and suppression of SNAI1 play critical roles in the conversion of MEFs into ciHeps. Overall, the high reprogramming efficiency and low safety risks of this method and the maturity of hepatocyte-like cells generated by this method make it an ideal approach to supply sufficient hepatocytes for various uses, such as cell transplantation therapies, disease modeling, and drug screening. Importantly, this method can reprogram MAFs into ciHeps. If it were proved effective in reprogramming human cells, then it could potentially provide an autologous cell source for future clinical applications to avoid immune rejection.

## Results

### Selection of small molecules for hepatic reprogramming of fibroblasts

In a previous study, we discovered that a small molecule combination consisting of SB431542, CHIR99021, BIX01294, and all-*trans*-retinoic acid (ATRA) could induce liver cancer cells to differentiate into hepatocyte-like cells while losing their malignant features (Zhang et al., 2022). ATRA is a well-known inducer of tumor cell differentiation, while the other chemicals in the combination, SB431542 [a transforming growth factor β (TGFβ) inhibitor], CHIR99021 (a Wnt activator), and BIX01294 (a histone methyltransferase inhibitor), are commonly used in cell reprogramming (Zhao, 2019). Interestingly, both TGFβ and Wnt signaling contribute to the long-term maintenance of the hepatocyte phenotype and function (Huch et al., 2015), and BIX01294 is commonly used to induce the expression of hepatocyte functional genes and stabilize the hepatocyte phenotype (Becker et al., 2017). Based on these findings, we hypothesized that SB431542, CHIR99021, and BIX01294 could be potential candidates for creating hepatocyte identity and converting cell fate.

After treating MEFs with these three molecules (SB431542, CHIR99021, and BIX01294) for 12 days, many cells acquired a polygonal morphology and some even possessed a binuclear structure, indicating the generation of hepatocyte-like cells (Supplementary Figure S1A). However, the conversion was only partial (Supplementary Figure S1A). Additionally, there were significantly fewer cells expressing albumin (ALB) and cytochrome P450 (CYP)3A, two hepatic markers, compared to mouse primary hepatocytes (MPHs) (Supplementary Figure S1B).

To investigate whether the observed phenomenon was due to the use of suboptimal molecule candidates, we tested other modulators of the same signaling pathway, including the TGFβ inhibitors A83-01 and RepSox, as well as the glycogen synthase kinase 3 (GSK3) inhibitors CHIR98014, LY2090314, and BIO (Supplementary Figure S2). However, their reprogramming efficiency, as assessed by cell morphology and ALB expression, was even lower than that of the three-factor combination of SB431542, CHIR99021, and BIX01294.

To enhance the reprogramming efficiency of the three-factor combination, we incorporated two additional promotional chemicals: LDN193189, a bone morphogenetic protein (BMP) inhibitor, and DAPT, a Notch inhibitor. These two chemicals have been shown to inhibit the expression of epithelial-to-mesenchymal transition (EMT)-related genes in previous studies (Xiang et al., 2019). Eventually, this five-factor SMC, consisting of SB431542, CHIR99021, BIX01294, LDN193189, and DAPT, significantly increased the conversion efficiency of MEFs into ciHeps. ciHeps induced by the five-factor SMC uniformly acquired a hepatocyte-like morphology (Supplementary Figure S3). Next, using this five-factor SMC, we systematically investigated the conversion process of MEFs into hepatic cells.

### SMC can induce the reprogramming of both MEFs and MAFs into ciHeps

**Figure 1:**
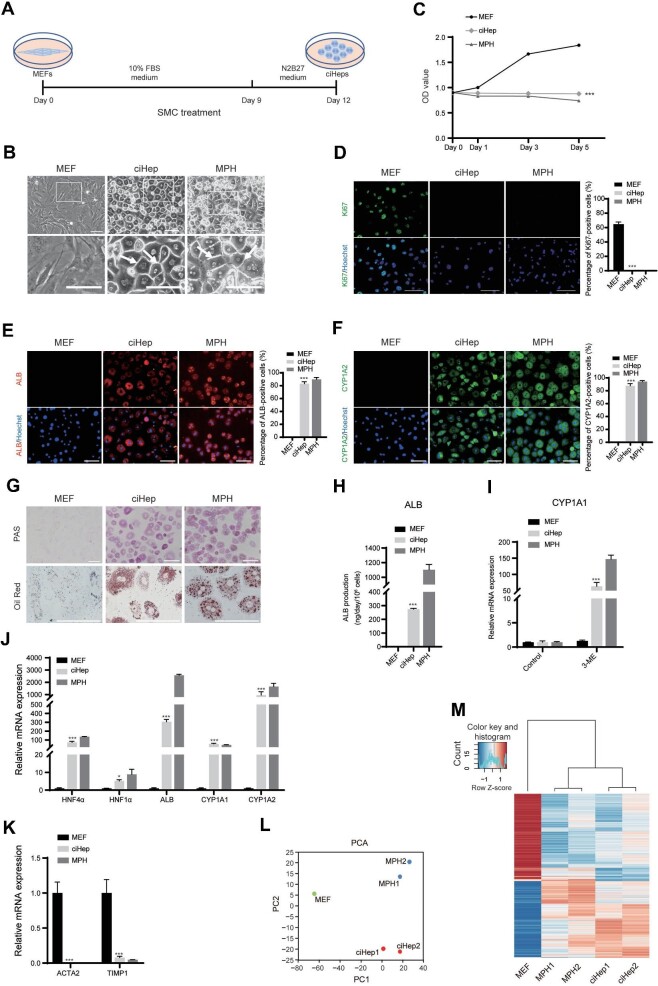
Reprogramming of MEFs into ciHeps under chemical induction. (**A**) Schematic diagram of chemical-induced direct reprogramming of MEFs into ciHeps. (**B**) Morphology of MEFs, ciHeps, and MPHs. (**C**) CCK8 assay revealing the cell growth abilities. (**D**) Immunofluorescence staining of Ki67 and the percentage of Ki67 positive cells. (**E** and **F**) Immunofluorescence staining of the hepatocyte markers ALB (**E**) and CYP1A2 (**F**) and the percentage of hepatocyte marker-positive cells. (**G**) PAS and Oil Red staining measuring the glycogen storage and lipid accumulation abilities of ciHeps. (**H**) ELISA measuring the ALB secretory function of ciHeps. (**I**) RT-qPCR assay showing drug (3-ME)-induced CYP1A1 activity of ciHeps after induction for 48 h. (**J** and **K**) RT-qPCR assay showing the increased expression levels of hepatic genes (**J**) and decreased expression levels of fibroblast-related genes (**K**) in ciHeps. (**L**) Two-dimensional PCA indicating that ciHeps resemble MPHs more closely than MEFs. (**M**) Global gene expression profiles demonstrating that the gene profiles of ciHeps are clustered with those of MPHs. Scale bar, 100 μm. *P < 0.05, ***P < 0.001.

MEFs treated with SMC for 12 days (Figure 1A) gradually transitioned from a mesenchymal morphology to a homogenous hepatocyte (epithelium) morphology. Some ciHeps exhibited a binuclear morphologic feature of mature hepatocytes (Figure 1B). The clear boundary-like structure of ciHeps resembled that of MPHs (Figure 1B, arrow), indicating that ciHeps acquired a bile duct structure (Reif et al., 2015; Korelova et al., 2019).

The Cell Counting Kit-8 (CCK8) assay results revealed that ciHeps, similar to MPHs, lost their proliferation ability *in vitro* (Figure 1C). Immunofluorescence staining of Ki67, a marker expressed in the nuclei of proliferating cells, showed that approximately 65% of the MEFs were Ki67-positive, whereas none of the ciHeps or MPHs were Ki67-positive (Figure 1D).

Immunofluorescence staining assays showed that ciHeps expressed hepatocyte-specific markers, such as ALB and CYP1A2. The percentages of ALB- or CYP1A2-positive ciHeps (83% and 88%, respectively) were comparable to those of MPHs (Figure 1E and F). These findings suggest that ciHeps acquired mature hepatocyte features.

In addition, positive Periodic Acid–Schiff (PAS) and Oil Red staining revealed that ciHeps gained glycogen and lipid accumulation functions (Figure 1G). Additionally, enzyme-linked immunosorbent assay (ELISA) demonstrated that ciHeps were able to secrete ALB (Figure 1H), which is crucial for cell transplantation therapy, as patients with chronic liver diseases often have impaired ALB synthesis in their native hepatocytes. Moreover, ciHeps showed increased CYP enzyme activity when exposed to drugs. Specifically, the activity of the CYP1A1 enzyme strikingly increased after 48 h of induction with 3-methylcholanthrene (3-ME) (Figure 1I).

Real-time fluorescent quantitative polymerase chain reaction (RT-qPCR) assay showed a significant increase in the expression level of hepatocyte-specific genes in ciHeps, including hepatocyte nuclear factor (HNF) 4α, HNF1α, ALB, CYP1A1, and CYP1A2 (Figure 1J). Meanwhile, the expression levels of fibroblast-related genes, including ACTA2 and TIMP1, were notably decreased in ciHeps (Figure 1K). The identity change was further confirmed by RNA-sequencing analysis in ciHeps. Principal component analysis (PCA) revealed that ciHeps more closely resembled MPHs than MEFs (Figure 1L). Global gene expression profiles showed that the gene profiles of ciHeps were clustered with those of MPHs (Figure 1M). However, they were distinctly different from those of MEFs.

Taken together, our study demonstrated that SMC treatment could indeed reprogram MEFs into ciHeps with mature hepatocyte morphology, biological behavior, functions, and gene expression patterns. The homogenous morphology and high expression of hepatocyte markers of ciHeps indicated the high efficiency of this approach.

To confirm that our protocol specifically generated ciHeps rather than other endoderm-derived cell types, we conducted an RT-qPCR assay to detect marker gene expression levels of other endoderm-derived cell types, such as pancreatic cells, intestinal epithelia, thyroid epithelia, and lung alveolar epithelial cells. The results revealed that SMC induction only resulted in the generation of ciHeps and did not produce any other endoderm-derived cell types (Supplementary Figure S4).

**Figure 2:**
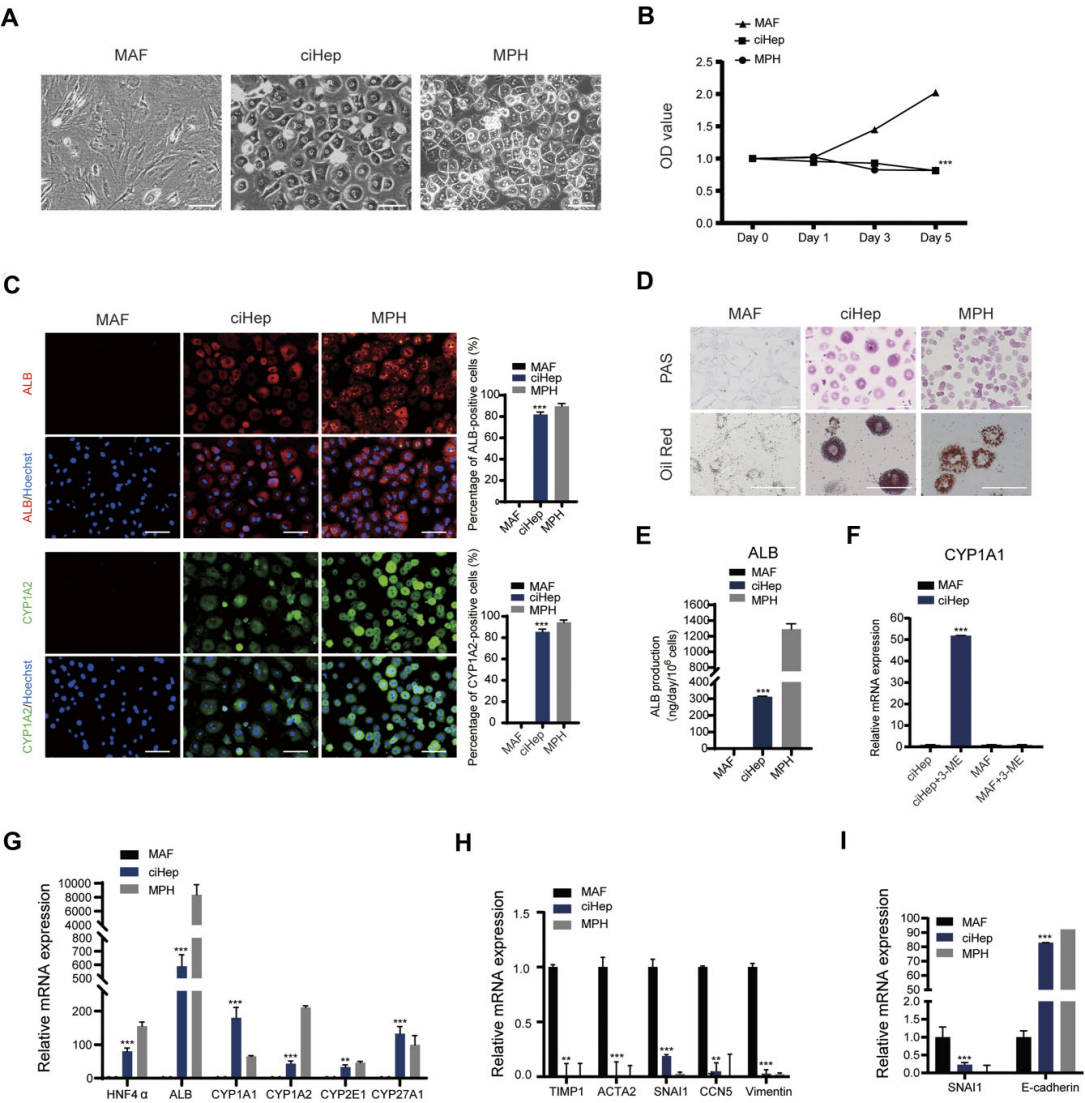
Reprogramming of MAFs into ciHeps under chemical induction. (**A**) Morphology of MAFs, MAF-derived ciHeps, and MPHs. (**B**) CCK8 assay revealing the cell growth abilities. (**C**) Immunofluorescence staining of the hepatocyte markers ALB and CYP1A2 and the percentage of hepatocyte marker-positive cells. (**D**) PAS and Oil Red staining measuring the glycogen storage and lipid accumulation abilities of MAF-derived ciHeps. (**E**) ELISA measuring the ALB secretory function of MAF-derived ciHeps. (**F**) RT-qPCR assay showing drug (3-ME)-induced CYP1A1 activity of MAF-derived ciHeps after induction for 48 h. (**G** and **H**) RT-qPCR assay showing the increased expression levels of hepatic genes (**G**) and decreased expression levels of fibroblast-related genes (**H**) in MAF-derived ciHeps. (**I**) RT-qPCR assay showing the expression levels of MET-related genes SNAI1 and E-cadherin in MAF-derived ciHeps. Scale bar, 100 μm. **P < 0.01, ***P < 0.001.

To further validate our results and the efficiency of this protocol, we isolated MAFs from mouse skin tissue and treated them with SMC. Similar to MEFs, MAFs were also converted into ciHeps with mature hepatocyte features (Figure 2). The high reprogramming efficiency of up to 80% suggested that autologous fibroblasts obtained from patients may be used to generate hepatocyte-like cells for clinical use in the future, thereby avoiding the risk of immune rejection.

### Chemical-induced direct reprogramming of MEFs into ciHeps bypassed intermediate stem cell and immature hepatoblast stages

**Figure 3:**
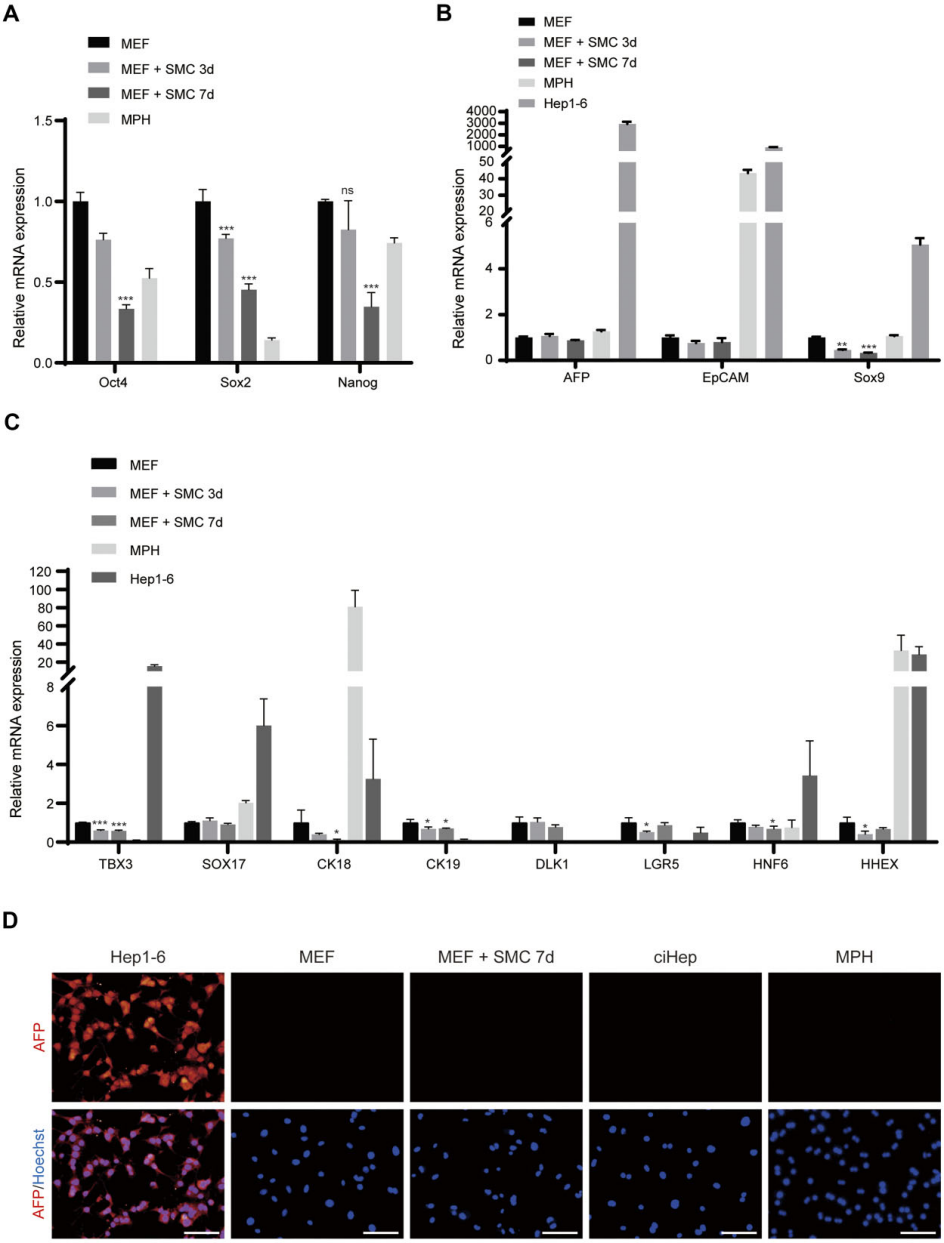
Direct reprogramming of MEFs into ciHeps bypasses intermediate stages. (**A**–**C**) RT-qPCR assay showing the expression levels of pluripotent genes (**A**), immature hepatoblast marker genes (**B**), and hepatic progenitor marker genes (**C**). (**D**) Immunofluorescence staining of the immature hepatoblast marker AFP. Scale bar, 100 μm. SMC 3d, SMC-treated cells on Day 3. SMC 7d, SMC-treated cells on Day 7. *P < 0.05, **P < 0.01, ***P < 0.001. ns, not significant.

To confirm that the ciHeps generated from MEFs were mature hepatic cells suitable for therapeutic use, we performed a series of experiments. First, we assessed the stemness and maturity of SMC-treated cells on Day 3 and Day 7 by examining the expression levels of pluripotency and immaturity genes. Elevated expression levels of pluripotency genes such as Oct4, Sox2, and Nanog were not detected in these cells (Figure 3A). We also examined the expression levels of immature hepatoblast marker genes, including α-fetoprotein (AFP), EpCAM, Sox9, and other hepatic progenitor marker genes in SMC-treated cells on Day 3 and Day 7. Our findings revealed that the expression levels of these genes did not increase during the reprogramming process in SMC-treated cells (Figure 3B and C). Furthermore, we found that mouse liver cancer cells Hep1-6 were positive for AFP, as determined by immunofluorescence staining, while no AFP expression was detected in ciHeps or SMC-treated cells on Day 7 (Figure 3D). Together, these results indicate that the fate conversion of MEFs into ciHeps by SMC induction was a direct process that bypassed the stem cell and immature hepatoblast stages, and the ciHeps generated were mature hepatic cells.

### MEF-derived ciHeps can engraft into the liver after transplantation

**Figure 4:**
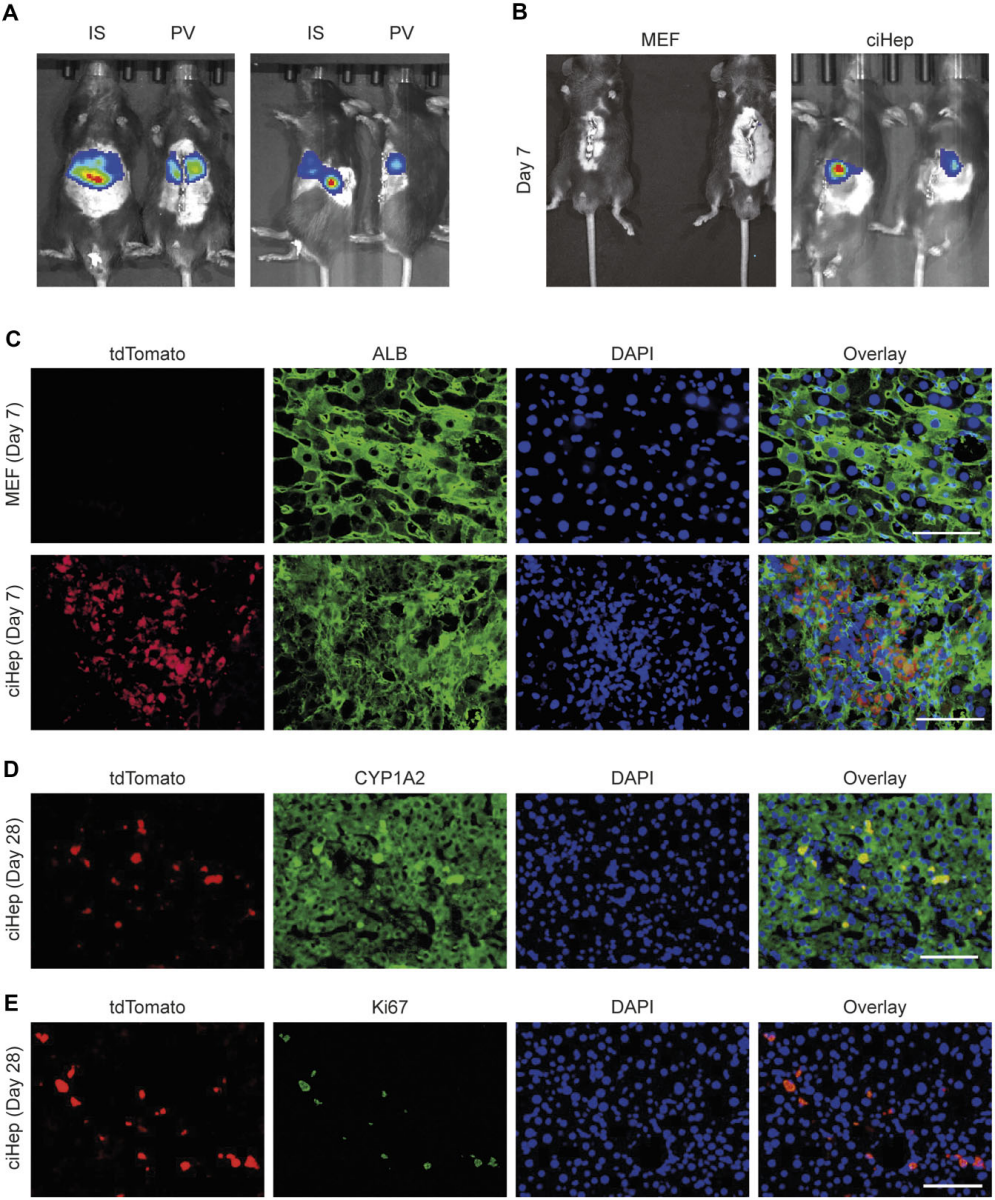
Engraftment and survival potential of MEF-derived ciHeps after transplantation. (**A**) Luciferase activities of ciHeps in mice transplanted via different routes. IS and PV represent intrasplenic and portal vein injection, respectively. The left panel is a supine image, and the right panel is a side sleeper image. (**B**) Luciferase activities of MEFs and ciHeps in mice 7 days after transplantation via portal vein injection. (**C**) Immunofluorescence staining of tdTomato and ALB in liver sections 7 days after MEF or ciHep transplantation. (**D** and **E**) Immunofluorescence staining of tdTomato (**D** and **E**), CYP1A2 (**D**), and Ki67 (**E**) in liver sections, 28 days after ciHep transplantation. Scale bar, 100 μm.

Next, we investigated the engraftment and survival potential of MEF-derived ciHeps to determine their therapeutic applicability. First, we generated ciHeps from MEFs isolated from EGFP-luciferase transgenic mice and transplanted them into C57BL/6J mice via intrasplenic or portal vein injection at a dose of 1 × 10^6^ or 0.3 × 10^6^ cells per mouse, respectively. By measuring luciferase activities, we found that cells infused via intrasplenic injection were primarily located in the liver and spleen (Figure 4A). The signal in the spleen was much stronger than that in the liver, indicating that most of the injected cells did not reach the liver. In contrast, cells infused via portal vein injection mostly reached the liver and remained there (Figure 4A). Therefore, portal vein injection is the preferred route of hepatocyte transplantation as it provides direct access to the hepatic sinusoids.

We injected MEFs and MEF-derived ciHeps into mice via the portal vein and monitored their survival *in vivo*. Seven days after injection, the signal of ciHeps was still detectable in the liver, while the signal of MEFs was not, indicating that ciHeps could engraft and survive in the liver (Figure 4B). For further validation, we generated ciHeps from MEFs isolated from mTmG transgenic mice and transplanted them into mice that had undergone 70% liver resection. ciHeps were injected into the portal vein at a dose of 0.3 × 10^6^ cells per mouse immediately after liver resection surgery. MEFs were used as a control. The livers were harvested 7 or 28 days after cell transplantation. Fluorescence images of the liver tissues revealed that a large number of ciHeps (tdTomato^+^ cells) were successfully engrafted into the liver. These cells stained positive for ALB, indicating that they retained the signature feature of hepatocytes (Figure 4C). In contrast, no tdTomato^+^ cells were observed in the control liver, indicating that MEFs were unable to survive in the liver (Figure 4C). After extending the observation period to 28 days, it was discovered that some tdTomato^+^ ciHeps still remained in the liver and were positive for CYP1A2 (Figure 4D). The presence of Ki67-positive signals indicated that the transplanted ciHeps had the ability to proliferate (Figure 4E). These results suggested that transplanted ciHeps could survive, engraft, and proliferate in the liver.

### Therapeutic effects of MEF-derived ciHep transplantation in mice with liver injuries

**Figure 5:**
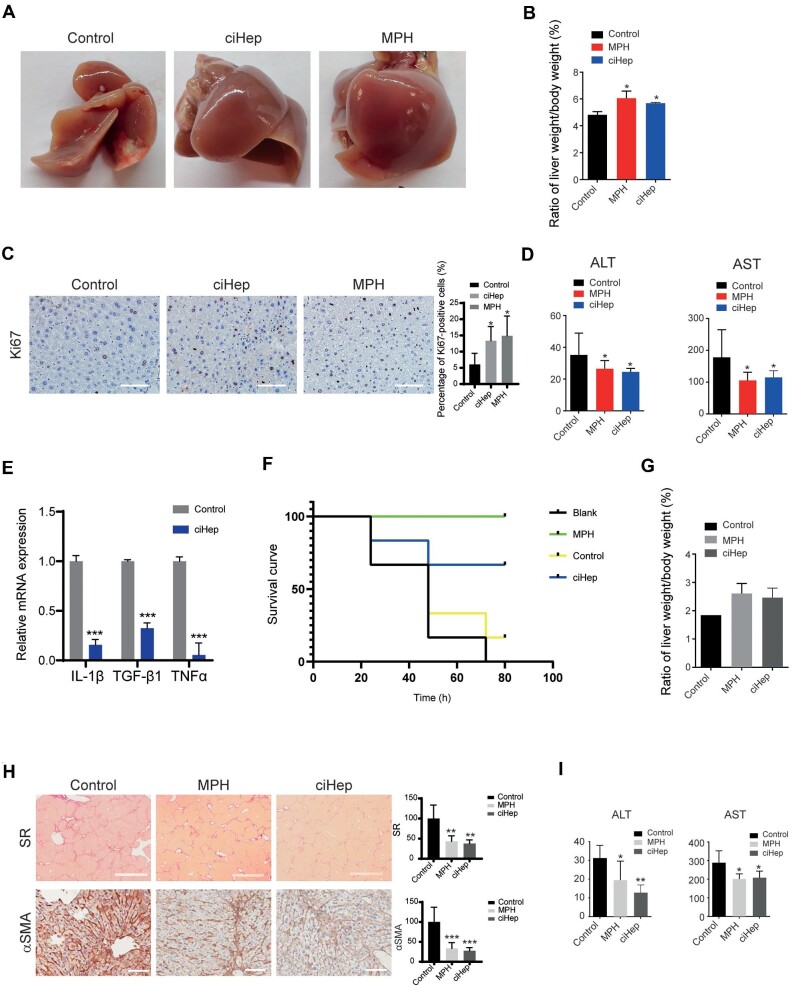
MEF-derived ciHeps exhibit therapeutic effects after liver injury. (**A**–**E**) MEF-derived ciHeps were transplanted into mice that underwent 70% liver resection. MEFs and MPHs were used as negative and positive controls, respectively. Mice were sacrificed 7 days after cell transplantation. (**A**) Images of the dissected livers. (**B**) The ratio of liver weight to body weight. (**C**) Immunohistochemical staining of Ki67 in liver sections. (**D**) Levels of serum ALT and AST. (**E**) RT-qPCR assay showing the expression levels of proinflammatory cytokines in liver tissues. (**F** and **G**) MEF-derived ciHeps were transplanted into mice that underwent 90% lethal liver resection. MEFs and MPHs were transplanted as negative and positive controls, respectively, while empty microcapsules served as the blank control. Surviving mice were sacrificed 72 h after cell transplantation. (**F**) The survival curve of mice. (**G**) The ratio of liver weight to body weight of surviving mice. (**H** and **I**) MEF-derived ciHeps were transplanted into CCl_4_-induced fibrotic mice. MPHs were transplanted as a positive control, and sterilizing saline was injected as a blank control. Mice were sacrificed 4 weeks after cell transplantation. (**H**) Immunohistochemical staining of SR and αSMA in liver sections. (**I**) Levels of serum ALT and AST. Scale bar, 100 μm. *P < 0.05, **P < 0.01, ***P < 0.001.

After confirming the engraftment and survival of MEF-derived ciHeps in the liver, we next investigated their potential therapeutic benefits in mouse models with liver injuries. MEF-derived ciHeps were transplanted via portal vein injection into mice that underwent 70% liver resection. MEFs and MPHs were used as negative and positive controls, respectively. Seven days after transplantation, we observed that the livers of mice that received ciHep injection were both larger in size and heavier in weight than those of mice that received MEF injection, and they were comparable to the livers of mice that received MPH injection (Figure 5A and B). Consistent with this observation, positive immunohistochemical staining of Ki67, a marker of proliferation ability, was extensively found in the ciHep-treated liver samples (Figure 5C). In addition, the levels of serum ALT and AST were significantly reduced in mice transplanted with ciHeps compared to those in control mice transplanted with MEFs (Figure 5D). These results suggested that ciHep transplantation could promote liver regeneration.

Liver injury can trigger hepatic inflammation, which is a prominent factor in disease progression. Thus, we examined the expression levels of the proinflammatory cytokines IL-1β, TGF-β1, and TNFα in harvested liver tissues and found that the levels of these cytokines were significantly reduced in mice transplanted with ciHeps (Figure 5E). This finding suggested that ciHep transplantation had an anti-inflammatory effect, which may help prevent the worsening of liver disease.

ACLF is a life-threatening disease with an extremely high mortality rate. According to Hernaez's report (Hernaez et al., 2019), 25.5% and 40.0% of patients with ACLF die within 1 month and 3 months of hospital admission, respectively. The 90% lethal liver resection mouse model mimics ACLF and provides a useful platform to test the *in vivo* functions of ciHeps under extreme conditions.

We transplanted 1 × 10^7^ MEF-derived ciHeps enwrapped in microcapsules into mice that underwent 90% lethal liver resection through intraperitoneal injection immediately after surgery. MEFs and MPHs were transplanted as negative and positive controls, respectively, while empty microcapsules served as the blank control. Intraperitoneal transplantation was chosen due to its ability to accommodate the large volume of cells required for therapeutic efficacy. Even though microencapsulated cells cannot engraft into mouse livers, they can perform critical functions and sustain the lives of mice when their own hepatocytes fail rapidly. As shown in Figure 5F, ciHep transplantation significantly elevated the cumulative survival rate of mice within 72 h (Figure 5F). Four out of six mice that received ciHeps survived, which was relatively close to the efficacy of MPH transplantation (all six mice survived). In contrast, only one out of six mice that received MEFs survived, and none of the mice that received empty microcapsules survived (Figure 5F).

After a 72-h period, we sacrificed the surviving mice and measured their liver weight and body weight. The ratio of liver weight to body weight revealed that ciHep transplantation promoted liver regeneration, with a comparable effect to that of MPH transplantation (Figure 5G). This indicated that ciHeps are indeed functional hepatocyte-like cells that can temporarily replace the role of hepatocytes in mice, thus reducing liver injury and promoting survival.

Chronic liver injuries can lead to fibrosis, a condition for which there is currently no effective therapeutic agent. To test the effect of ciHep transplantation on liver fibrosis, we used the carbon tetrachloride (CCl_4_)-induced fibrotic mouse model and transplanted 0.3 × 10^6^ MEF-derived ciHeps via the portal vein. An equal amount of MPH transplantation served as a positive control, and sterilizing saline injection served as a blank control. We continued administering CCl_4_ every 3 days for 4 weeks after cell transplantation to prevent spontaneous fibrosis regression.

Four weeks after cell transplantation, mice that received ciHep transplantation showed reduced liver fibrosis, as indicated by reduced Sirius Red (SR) and alpha-smooth muscle actin (αSMA) immunohistochemical staining (Figure 5H). In addition, mice that received ciHep transplantation showed decreased levels of serum ALT and AST, indicating an improvement in liver function (Figure 5I). These results suggested that ciHep transplantation can effectively reduce liver fibrosis and improve liver function, with a level of effectiveness comparable to that of MPH transplantation.

### MEF-derived ciHeps are a safe cell source for transplantation

**Figure 6:**
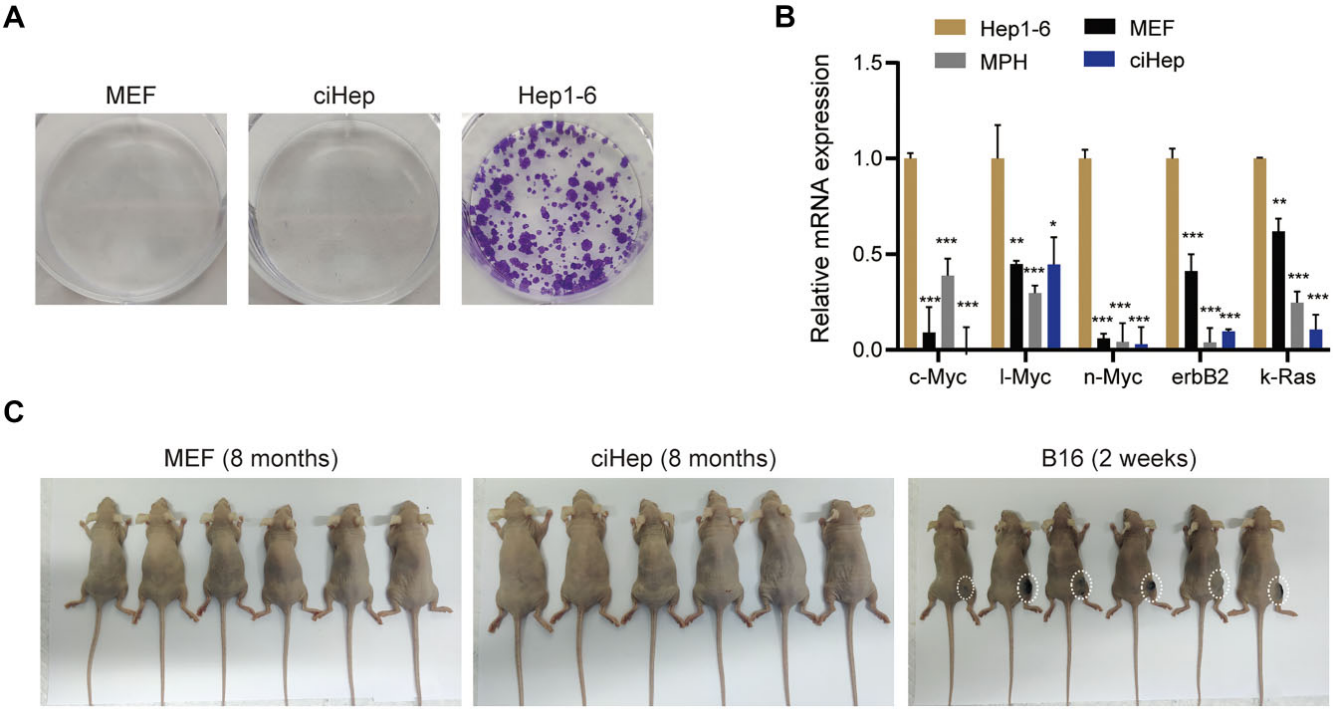
MEF-derived ciHeps show no tumorigenic potential. (**A**) *In vitro* colony formation assay. (**B**) RT-qPCR assay showing the expression levels of oncogenes. (**C**) *In vivo* subcutaneous xenograft study. *P < 0.05, **P < 0.01, ***P < 0.001.

We have demonstrated that the transplantation of MEF-derived ciHeps has therapeutic efficacy after liver injury. Then, we proceeded to investigate the safety of this cell source. The colony formation assay showed that neither ciHeps nor MEFs formed cell clones (Figure 6A). As a positive control, a large number of clones were formed by the liver cancer cell line Hep1-6 (Figure 6A). This suggested that ciHeps did not have tumorigenic potential *in vitro*. We also tested the expression levels of oncogenes, such as c-Myc, l-Myc, n-Myc, erbB2, and k-Ras, and found that these genes were not activated in ciHeps (Figure 6B), which is consistent with the results of the colony formation assay.

We then tested the tumorigenic potential of MEF-derived ciHeps *in vivo* through a subcutaneous xenograft study. MEFs and the melanoma cell line B16 were used as negative and positive controls, respectively. Each group consisted of six mice injected with 1 × 10^6^ cells per mouse. Within the 2-week observation window, we found the emergence of tumor nodules at the inoculation sites of B16 cells but not where ciHeps or MEFs were injected (Figure 6C). We extended the observation window to 8 months and still did not observe tumor formation in mice injected with ciHeps or MEFs (Figure 6C). Together, these results demonstrated that MEF-derived ciHeps were a safe cell source with no tumorigenic potential either *in vitro* or *in vivo*.

### SMC-induced MET and suppression of SNAI1 contribute to the fate conversion of MEFs into ciHeps

**Figure 7:**
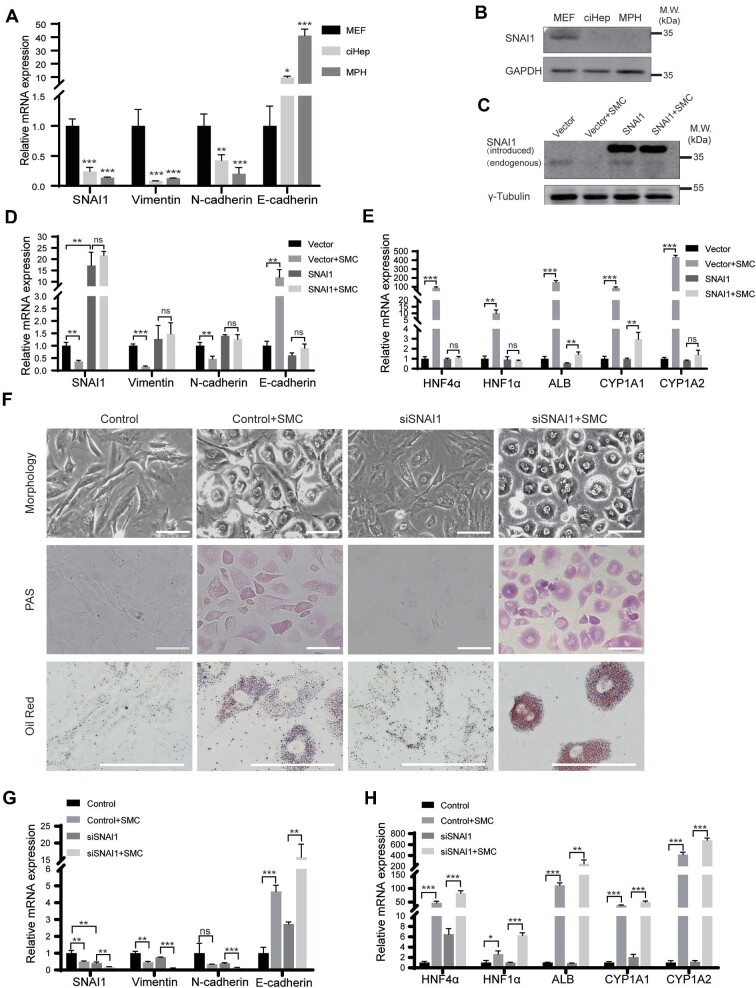
SMC-induced MET and suppression of SNAI1 promote cell fate conversion. (**A**) RT-qPCR assay showing the expression levels of MET-related genes in MEFs, ciHeps, and MPHs. (**B**) Western blot analysis showing the expression levels of SNAI1 in MEFs, ciHeps, and MPHs. (**C**) Western blot analysis showing the expression levels of SNAI1 in control or SNAI1-overexpressing MEFs with or without SMC treatment for 12 days. (**D** and **E**) RT-qPCR assay showing the expression levels of MET-related genes (**D**) and hepatocyte-specific genes (**E**) in control or SNAI1-overexpressing MEFs with or without SMC treatment for 12 days. (**F**) Morphology and PAS and Oil Red staining of control or SNAI1-knockdown MEFs with or without SMC treatment for 8 days. (**G** and **H**) RT-qPCR assay showing the expression levels of MET-related genes (**G**) and hepatocyte-specific genes (**H**) in control or SNAI1-knockdown MEFs with or without SMC treatment for 8 days. Scale bar, 100 μm. *P < 0.05, **P < 0.01, ***P < 0.001. ns, not significant.

In this study, we investigated the role of SMC in the fate conversion of fibroblasts into ciHeps, which involves the critical process of MET (Li et al., 2010; Samavarchi-Tehrani et al., 2010). Our results showed that SMC treatment induced the upregulation of E-cadherin, a marker of the epithelial phenotype. Meanwhile, the expression levels of the mesenchymal phenotype markers SNAI1, Vimentin, and N-cadherin were strikingly suppressed. These findings suggested that SMC treatment promoted the MET cascade during the conversion of MEFs into ciHeps (Figure 7A).

SNAI1 is a crucial signaling node in the EMT process and plays an essential role in the regulation of hepatocyte morphogenesis and differentiation. This is achieved by suppressing the expression of HNF4α, which is the master gene responsible for constructing hepatocyte identity (Cicchini et al. 2006; Noce et al. 2019). We found that SNAI1 expression was significantly repressed in ciHeps (Figure 7B). This finding, combined with the previously observed high expression of HNF4α, suggested that the suppression of SNAI1 by SMC may contribute to the fate conversion of MEFs into ciHeps.

To further demonstrate the importance of SNAI1 in the reprogramming of MEFs, we utilized lentivirus infection to overexpress SNAI1 in MEFs (Figure 7C) and subsequently treated these cells with SMC for 12 days. We found that the externally introduced SNAI1 protein remained unaffected by SMC treatment (Figure 7C), and the expression levels of MET-related genes such as Vimentin and N-cadherin were not suppressed (Figure 7D). Accordingly, we observed no upregulation in the expression levels of hepatocyte-specific genes in SNAI1-overexpressing MEFs (Figure 7E). In contrast, knocking down SNAI1 expression in MEFs using SNAI1 siRNA significantly improved the transformation efficiency of these cells into ciHeps (Figure 7F–H). Interestingly, SNAI1 knockdown promoted the expression of HNF4α. However, the beneficial effect of SNAI1 knockdown on the expression of other hepatocyte genes, such as ALB and CYP1A2, was minimal, and SNAI1 knockdown had little to no impact on HNF1α, another important nuclear transcription factor for liver cell lineage determination (Figure 7H).

Overall, our results suggest that the suppression of SNAI1 by SMC not only supports the MET of MEFs by repressing EMT but also promotes MEFs to acquire hepatocyte characteristics by relieving the suppression of HNF4α, a master gene responsible for constructing hepatocyte identity. However, successful reprogramming of MEFs into ciHeps involves multiple mechanisms, which rely on the synergistic effects of the five-factor SMC. For example, we observed the activation of several transcription factors, including HNF4α, HNF1α, GATA4, FOXA1, and FOXA3, at different stages in MEFs treated with SMC (Supplementary Figure S5). Clarifying all the underlying mechanisms requires further investigation, but our study does provide valuable insights into the complex process of cell fate conversion and the critical role of SNAI1 in this process.

## Discussion

In this study, we report a novel and highly efficient method for directly reprogramming fibroblasts into hepatocyte-like cells using small molecules as the sole inducers. This approach bypasses intermediate stem cell and immature hepatoblast stages and generates homogeneous, mature, and functional hepatic cells in one step. The ciHeps generated can not only engraft into the liver and survive but also perform critical hepatocyte functions, thus promoting liver regeneration and improving survival rates in mice with liver injuries. Moreover, ciHeps are a safe cell source with no tumorigenic risk either *in vitro* or *in vivo*, which makes them highly suitable for therapeutic use. Mechanistically, SMC-induced MET and suppression of SNAI1 activity contributed to the successful reprogramming of fibroblasts into ciHeps.

Hepatocyte transplantation is considered a potential therapeutic approach for patients suffering from liver diseases, especially those with congenital metabolic disorders. However, the restricted supply of primary hepatocytes has led to the exploration of alternative cell sources, such as fetal hepatocytes, immortalized hepatocytes, mesenchymal stem cells, fibroblast-derived hepatocyte-like cells, and pluripotent stem cell-derived hepatocytes. Despite their potential, none of these alternatives have been adopted in clinical practice due to their inherent limitations. The efficient generation of mature and functional hepatocyte-like cells for cell transplantation therapy remains a challenge. Our study presents a new method for generating mature and functional hepatocyte-like cells, called ciHeps, from fibroblasts using a chemical induction approach. The advantages of this method include (i) ciHeps possess similar features and functions as mature hepatocytes, and they can engraft into and repopulate the liver; (ii) fibroblasts are easy to obtain and can be rapidly expanded to provide large cell quantities; (iii) autologous fibroblasts can be used for conversion into ciHeps to avoid the risk of immune rejection; (iv) ciHeps are fully differentiated, somatic-derived cells that are reprogrammed through chemical induction rather than genetic manipulation, thereby reducing the risk of tumorigenicity; and (v) the ciHep reprogramming protocol is simple, inexpensive, and highly efficient (with a conversion rate of over 80%). In summary, if this chemical-induced reprogramming method also proved effective in human cells, then the ciHeps generated could potentially be a valuable cell source for clinical use in the future.

In the present study, the selected molecules are SB431542, CHIR99021, BIX01294, LDN193189, and DAPT, which play different roles in the reprogramming process. SB431542, for example, inhibits the TGFβ signaling pathway and promotes the process of MET during cell reprogramming (Ichida et al., 2009). CHIR99021, a GSK3 inhibitor, can activate the Wnt pathway and regulate cell metabolism (Xie et al., 2017; Zhao, 2019). By phosphorylating substrates, GSK3β can regulate a variety of metabolic pathways besides glycogen metabolism (Picton et al., 1982; Potapova et al., 2000; Jellusova et al., 2017). It is interesting to note that regulators of TGFβ and GSK3/Wnt signaling pathways are not only commonly used in reprogramming studies but also in primary hepatocyte culture. Previous studies have reported that regulating both the TGFβ and GSK3/Wnt signaling pathways could contribute to the phenotypic and functional maintenance of hepatocytes over the long term (Huch et al., 2015; Xiang et al., 2019). Inhibition of the TGFβ and GSK3/Wnt signaling pathways could also facilitate HNF1α-induced hepatic reprogramming (Lim et al., 2016). These results indicate that both signaling pathways are involved in the master gene regulatory network of hepatocytes. BIX01294 was used to overcome the ‘epigenetic barrier’ in the reprogramming process (Hormanseder, 2021; Zeng et al., 2021) by inhibiting histone methyltransferase. BIX01294 is also able to promote the expression of hepatocyte-related genes by demethylating histones (Becker et al., 2017). LDN193189 and DAPT inhibited the BMP and Notch signaling pathways, respectively, which can help inhibit the EMT process (Xiang et al., 2019) to enhance the efficiency of reprogramming and accelerate the process. LDN193189 and DAPT also have beneficial effects on maintaining primary hepatocyte identity *in vitro* (Xiang et al., 2019). Thus, through synergistic effects, our selected SMC effectively converts MEFs into ciHeps.

As many studies have demonstrated, MET is a key barrier that needs to be overcome during cell reprogramming (Li et al., 2010; Samavarchi-Tehrani et al., 2010). SNAI1 is a key node in the EMT–MET process that can bind to E-box DNA sequences and repress epithelial genes (Peinado et al., 2007; Xu et al., 2009). SNAI1 also affects hepatocyte differentiation by directly binding to the promoter of HNF4α and suppressing its expression (Cicchini et al., 2006; Noce et al., 2019). The expression of SNAI1 can be activated through the cooperation of multiple signaling pathways, including TGFβ, BMP, Notch, etc. (Peinado et al., 2007). In our studies, SB431542, LDN193189, and DAPT directly inhibited these signaling pathways and synergistically suppressed SNAI1 expression, thus enhancing the MET process.

Despite these encouraging results, several questions still need to be addressed before this method can be clinically applied. For example, it is unclear whether this chemical-induced reprogramming method will be as effective on human fibroblasts as it was on mouse fibroblasts or whether any modifications will be necessary. Additionally, ciHeps cultured *in vitro* lose their proliferation ability, much like primary hepatocytes. Therefore, producing ciHeps on a large scale to meet the demand for cell transplantation remains a challenge. Despite these challenges, this study provides a novel approach for generating functional hepatocyte-like cells that is safe, less complex, and cost-effective. If proved successful in human fibroblasts, this method may provide a safe alternative hepatocyte source for regenerative medicine, tissue engineering, pharmaceutical research, and disease modeling.

## Materials and methods

### Ethics approval

The animal use and care in this study were approved by the Animal Care and Use Committee of the corresponding author's institution. The mice were randomly allocated into different groups.

### Isolation and culture of MEFs, MAFs, and MPHs

MEFs were isolated from C57BL/6J (GemPharmatech Co., Ltd) mouse embryos at embryonic day 13.5 (E13.5). The head, tail, limbs, and internal organs were removed, and the remaining tissues were cut into pieces and trypsinized to obtain single-cell suspensions. MEFs were cultured in Dulbecco's modified Eagle's medium (DMEM, Biological Industries) supplemented with 10% fetal bovine serum (FBS, Biological Industries), 100 units/ml penicillin (Gibco), and 100 μg/ml streptomycin (Gibco) at 37°C with 5% CO_2_. The third to fifth passages of MEFs were utilized for reprogramming induction. MAFs were isolated from C57BL/6J mice (8 weeks old, GemPharmatech Co., Ltd). Adult mice were sacrificed, shaved, and then smeared with iodine volts and 75% alcohol three times and washed with phosphate buffered saline (PBS, HyClone) three times. The back skin of the mice was dissected and treated with 0.25% trypsin for 2 h. Then, the epidermis was removed, leaving only the dermis. After washing with PBS, the dermis was cut into pieces and further trypsinized with type I collagenase (1 mg/ml, Worthington) at 37°C for 2 h. The resulting cell suspension was filtered through a 70-μm cell strainer (Falcon) to obtain a single-cell suspension. After centrifugation, the cells were cultured in DMEM supplemented with 10% FBS, 100 units/ml penicillin, and 100 μg/ml streptomycin at 37°C with 5% CO_2_. MPHs were isolated from C57BL/6J mice using a previously established protocol (Li et al., 2010). Briefly, MPHs were seeded onto rat tail collagen-coated plates and cultured in DMEM supplemented with 10% FBS for 4 h. Once the cells adhered to the plates, the medium was replaced with DMEM supplemented with 0.5% N2 (Basal Media) and 1% B27 (Basal Media) to maintain the MPHs.

### Generation of ciHeps

MEFs or MAFs were seeded onto six-well plates and then treated with SMC to generate ciHeps. The reprogramming process was carried out in two distinct stages. The cell reprogramming medium (CRM) used in stage I was composed of DMEM supplemented with 10% FBS, 100 units/ml penicillin, 100 μg/ml streptomycin, 2 μM SB431542, 0.5 μM LDN193189, 2 μM BIX01294, 3 μM CHIR99021, and 2 μM DAPT. This medium was changed every 3 days. After a 9–12-day period, the cells were cultured in fresh medium. The CRM used in stage II was composed of DMEM supplemented with 100 units/ml penicillin, 100 μg/ml streptomycin, 5 μM SB431542, 0.5 μM LDN193189, 2 μM BIX01294, 4 μM CHIR99021, 2 μM DAPT, 0.5% N2, and 1% B27. After stage II treatment for 3–6 days, fibroblast-derived ciHeps emerged. The small molecules used in the experiment were purchased from Selleckchem. For more information about the SMC, please refer to Supplementary Table S1.

### ALB secretion, PAS staining, Oil Red staining, and CYP1A1 drug induction

To analyze ALB secretion, cells were cultured for 24 h, after which the supernatants were collected. The Mouse Albumin ELISA Kit (Bethyl Laboratory) was used to detect ALB following the manufacturer's instructions. For PAS staining and Oil Red staining, cells were fixed with 4% paraformaldehyde for 10 min at room temperature and washed with PBS. Staining was performed using the PAS Kit (Sigma) or Oil Red Kit (Sigma) following the manufacturer's instructions. For CYP1A1 drug induction, cells were treated with 3-ME for 48 h, and RT-qPCR was performed to analyze the gene expression levels of CYP1A1 as described above.

### Cell tracing

EGFP-luciferase-labeled cells were transplanted into C57BL/6J mice via intrasplenic or portal vein injection at a dose of 1 × 10^6^ or 0.3 × 10^6^ cells per mouse, respectively. The location of the cells was monitored on the specified days via *in vivo* imaging based on luciferase activity.

### For 70% liver resection and cell transplantation

Anesthetized mice underwent a 70% liver resection procedure, during which the left lateral lobe and the median lobe were removed. Transplantation of MEFs, ciHeps, and MPHs at a dose of 0.3 × 10^6^ cells per mouse via portal vein injection was performed immediately after 70% liver resection. Seven days after cell transplantation, the recipient mice were sacrificed, and blood and liver samples were collected for further analysis.

### For 90% lethal liver resection and generation of microencapsulated cells

Anesthetized mice underwent a 90% lethal liver resection procedure, during which the right lobes, median lobe, and left lateral lobe were removed, leaving only the two caudate lobes (posterior caudate lobe and anterior caudate lobe) intact. To generate microencapsulated cells, the static electricity drip technique was employed to create alginate-poly-L-lysine-alginate (APA) microcapsules containing MEFs, ciHeps, and MPHs. Briefly, cells were suspended in filter-sterilized saline containing 1.5% (*w*/*v*) sodium alginate solution (Sigma) at a final cell density of 10 × 10^6^ cells/ml. Fresh MPHs were used for the experiment. Then, the cell suspension was extruded into a 100 mM CaCl_2_ solution through a 0.4-mm needle using an electrostatic droplet generator, and calcium alginate gel beads formed. After washing three times, the calcium alginate gel beads were incubated with 0.05% (*w/v*) poly-L-lysine (molecular weight 21900; Sigma–Aldrich) to form an alginate-poly-L-lysine membrane around the surface. After washing the beads with saline three times, 0.15% (*w/v*) alginate was used for further coating. The beads were then suspended in 55 mM sodium citrate to make the alginate gel core liquefied. Finally, APA microcapsules with a diameter of approximately 350 μm were generated and used for transplantation.

### CCl_4_-induced liver fibrosis and cell transplantation

Six-week-old C57BL/6J mice were obtained from GemPharmatech Co., Ltd. Nanjing. To generate a liver fibrosis model, mice were injected with 0.25 μl/g CCl_4_ (Sinopharm Group Chemical Reagent Co. Ltd) dissolved in olive oil (Sinopharm Group Chemical Reagent Co. Ltd) through intraperitoneal injection for 8 weeks (three injections per week). After this, the mice were randomly allocated into different groups for cell transplantation. Then, 0.3 × 10^6^ ciHeps or MPHs were injected via the portal vein. Sterilizing saline was injected as a blank control. CCl_4_ was administered every 3 days during the 4-week period after transplantation to prevent fibrosis regression. Four weeks after cell transplantation, the recipient mice were sacrificed, and blood and liver samples were collected for further analysis.

### Statistical analysis

All data are expressed as mean ± SD. Statistical analysis was performed by the Prism software (GraphPad). The significance of differences between two groups was estimated by unpaired Student's *t*-test, while the significance of differences among multiple groups was determined by one-way analysis of variance. A *P*-value < 0.05 was considered statistically significant.

## Supplementary Material

mjad071_Supplemental_File

## Notes

**Author contributions:** Z.Z., J.D., X.Z., T.G., and Y.H. performed the experiments. Z.Z. and J.D. analyzed the data. Z.Z. wrote the manuscript. P.Z. designed the project and revised the manuscript. P.Z. and H.W. supervised the research work.
