# Supplementary material for: Highly efficient conversion of mouse fibroblasts into functional hepatic cells under chemical induction
Source: J Mol Cell Biol. 2023 Nov 23;15(11):mjad071. doi: 10.1093/jmcb/mjad071 (PMC11121195; doi:10.1093/jmcb/mjad071)
Supplement: mjad071_Supplemental_File [file mjad071_supplemental_file.pdf]

Highly Efficient Conversion of Mouse Fibroblasts into Functional Hepatic Cells under Chemical Induction

Supplementary Information

Zhi Zhong[1, 2], Jiangchuan Du [2], Xiangjie Zhu [2,3], Lingting Guan[2], Yanyu Hu[2], Peilin Zhang [2,*], and Hongyang Wang[1,2,*]

1. Fudan University Shanghai Cancer Center, Department of Oncology, Shanghai Medical College, Fudan University, Shanghai 200032, China

2. National Center for Liver Cancer, Naval Medical University, Shanghai 201805, China.

3. Institute of Metabolism & Integrative Biology, Fudan University, Shanghai 200438, China

* These authors jointly supervised this work.

Supplementary Materials and Methods

RT-qPCR

Total RNA was extracted using the TRIzol method, following the manufacturer's instructions. Subsequently, cDNA was synthesized from 1 μg of total RNA using the RevertAid First Strand cDNA Synthesis Kit (Thermo Scientific) according to the manufacturer's protocol. RT-qPCR was performed and analyzed on a LightCycler 480 II (Roche) using SYBR Green Master Mix (Vazyme) for relative quantification of the targeted genes. The primer sequences used in this study are listed in Supplementary Table S2.

Immunofluorescence staining

Cells were fixed with 4% paraformaldehyde (Sigma) for 10 min at room temperature and washed with PBS three times. Liver tissues were fixed with 4% paraformaldehyde for 24 h at 4°C, followed by a 30% sucrose solution for 12 h at 4°C. Tissue sections were obtained using a freezing microtome (Leica). Cells or liver tissue sections were permeabilized with 0.3% Triton X-100 (Sigma) in PBS for 15 min at room temperature. After being blocked with 10% normal goat serum (NGS, Sigma) and 1% bovine serum albumin (BSA, Sigma) in PBS for 1 h at room temperature, cells or sections were incubated with primary antibodies overnight at 4°C and washed with PBS three times. Finally, secondary antibodies conjugated with fluorescence were incubated for 30 min at room temperature in the dark. Nuclei were stained with Hoechst 33342 (Sigma). Secondary antibodies were AMCA AffiniPure Goat Anti-Rabbit IgG (H+L) (Cat# 111-155-045, Jackson ImmunoResearch Laboratories, Inc., 1:300). Primary antibodies were rabbit anti-ALB antibody (Cat# ab207327, Abcam, 1:200), rabbit anti-CYP1A2 antibody (Cat# ab170204, Abcam, 1:100), rabbit anti-AFP antibody (Cat# 14550-1-AP, Proteintech, 1:200), and rabbit anti-Ki67 antibody (Cat# ab16667,

Abcam, 1:250).

Fluorescence-activated cell sorting (FACS) cytometry

Cells were fixed with 4% paraformaldehyde for 10 min at room temperature and then washed with PBS three times. Subsequently, cells were blocked with 10% NGS containing 0.1% Triton X-100 for 10 min at room temperature. After centrifugation, cells were incubated with primary antibodies for 1 h at room temperature and then washed with PBS three times. The cells were then incubated with appropriate secondary antibodies for 30 min at room temperature in the dark. Following incubation, cells were washed again and analyzed using FACS analysis. The primary antibody isotype was used as a negative control. Primary antibodies were rabbit anti-HNF4α antibody (Cat# ab201460, Abcam, 1:200), rabbit anti-ALB antibody (Cat# ab207327, Abcam, 1:200), mouse anti-AAT (Cat# MA5-14661, Invitrogen, 1:50), and rabbit anti-CYP3A antibody (Cat# PA5-14896, Invitrogen, 1:50).

CCK8 assay

Cells were dispensed into 96-well plates with each well containing 1×10^4 cells. Cell proliferation was assessed by the CCK8 assay (APExBIO) following the manufacturer's instructions at the specified time. The optical density (OD) was recorded at 450 nm.

Serum biochemistry

An automatic biochemical analyzer (Mindray) was used to measure the levels of serum ALT and AST.

Histology and immunohistochemistry

Liver tissues were fixed overnight with 10% formalin (Sigma) and embedded in paraffin. Tissue sections, 5 μm in thickness, were obtained for further assays. The Picro Sirius Red Stain Kit (Abcam)

was used to stain the tissue sections for Sirius Red staining, following the manufacturer's instructions. For immunohistochemical staining, tissue sections were treated with 3% H_2O_2 for 20 min. The sections were then blocked in PBS containing 1% BSA for 30 min at 37°C. Sections were stained with primary antibodies at 4°C overnight. This was followed by incubation with secondary antibodies (Jackson ImmunoResearch Laboratories, Inc.) for 30 min at 37°C and DAB staining (Dako). Primary antibodies were rabbit anti-Ki67 antibody (Cat# ab16667, Abcam, 1:1000) and rabbit anti-αSMA (Cat# ab5694, Abcam, 1:1000).

Plate colony formation assay and subcutaneous xenograft study

For the plate colony formation assay, 1000 live MEFs, ciHeps, or mouse liver tumor cells were seeded into 6-well plates and cultured for 15 days. Then, the cells were fixed and stained with crystal violet. For the subcutaneous xenograft study, approximately 1×10^6 MEFs, ciHeps, or mouse melanoma cells were suspended in 50 μl DMEM containing 50% Matrigel (BD Biosciences). Cells were then injected subcutaneously into the right leg of nude mice. Tumor formation was monitored at the indicated times.

Lentivirus infection

The plasmid expressing SNAI1 was obtained from OBiO Technology (Shanghai) Corp. A control vector, which was an empty plasmid, was also used. To establish SNAI1-overexpressing MEFs, a lentiviral vector system was utilized.

RNA interference

siRNAs were obtained from OBiO Technology (Shanghai) Corp. A siRNA that did not contain any sequences against SNAI1 was used as a negative control. For RNA interference, siRNAs were transfected using Lipo8000 Transfection Reagent (Beyotime) at a final concentration of 50 nM

according to the manufacturer’s instructions.

Western blot

The whole-cell lysates were separated on 10% SDS-polyacrylamide gels and then transferred to nitrocellulose membranes (GE). The membranes were blocked using 5% BSA for 2 h at room temperature and then incubated with primary antibodies overnight at 4°C. Thereafter, the membranes were incubated with a fluorescein-conjugated secondary antibody for 1 h at room temperature. A fluorescent infrared signal was detected by the LI-COR Odyssey imaging system. Primary antibodies are as follows: rabbit anti-SNAI1 antibody (Cat# 3879, Cell Signaling Technology, 1:1000), rabbit anti-GAPDH antibody (Cat# AC001, ABclonal, 1:10000), and rabbit anti-γ-Tubulin antibody (Cat# ab16504, Abcam, 1:1000). The secondary antibody is IRDye 800CW goat anti-rabbit IgG (LI-COR).

Supplementary Figure

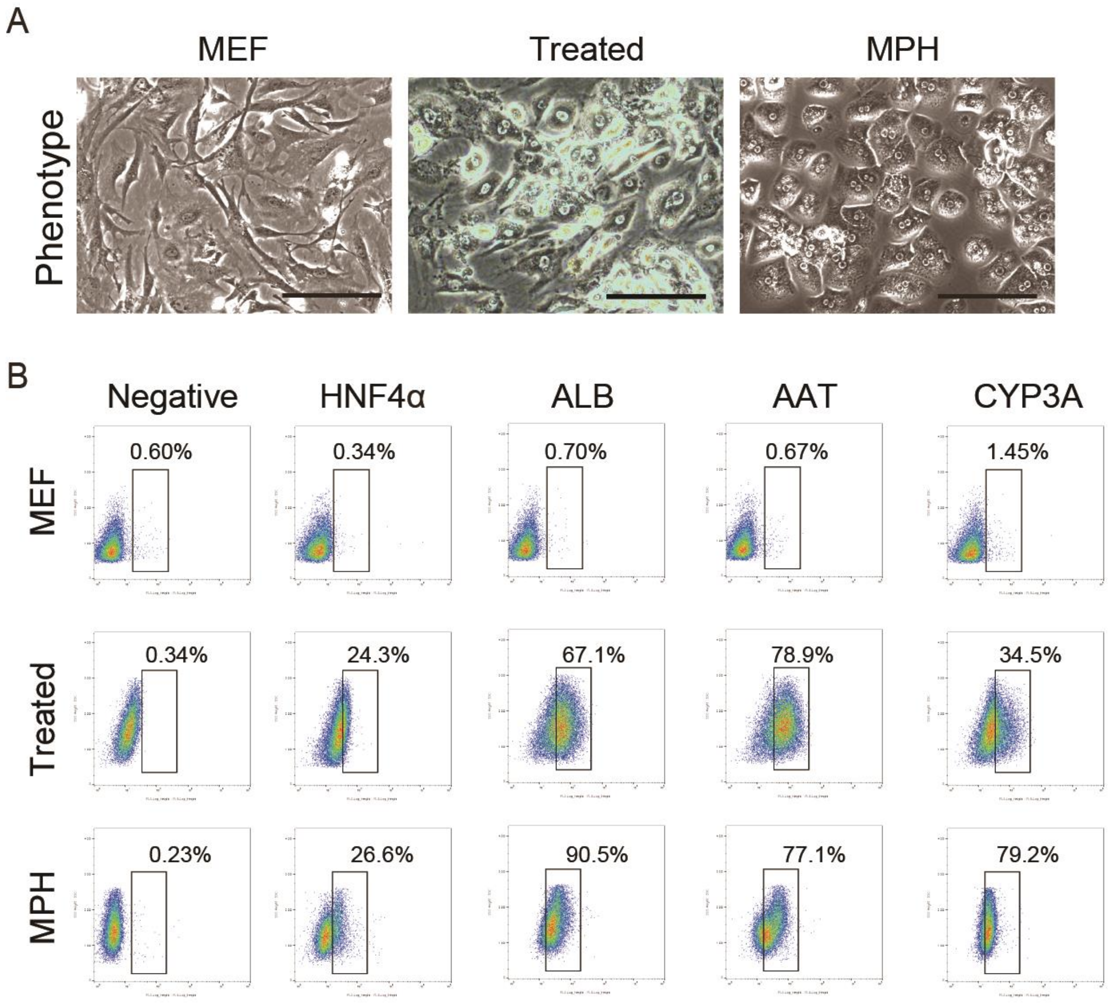

Supplementary Figure S1. Screening experiment for small molecules for hepatic reprogramming of fibroblasts. (**A**) Phase-contrast images of MEFs, 3 factor (SB431543, CHIR99021, BIX01294)-treated MEFs on day 12 of culturing. MPHs cultured overnight were used as positive controls. (**B**) FACS analysis results showing percentage of cells staining positive for hepatocyte markers in MEFs, 3 factor-treated MEFs and MPHs. All scale bar, 100 μm.

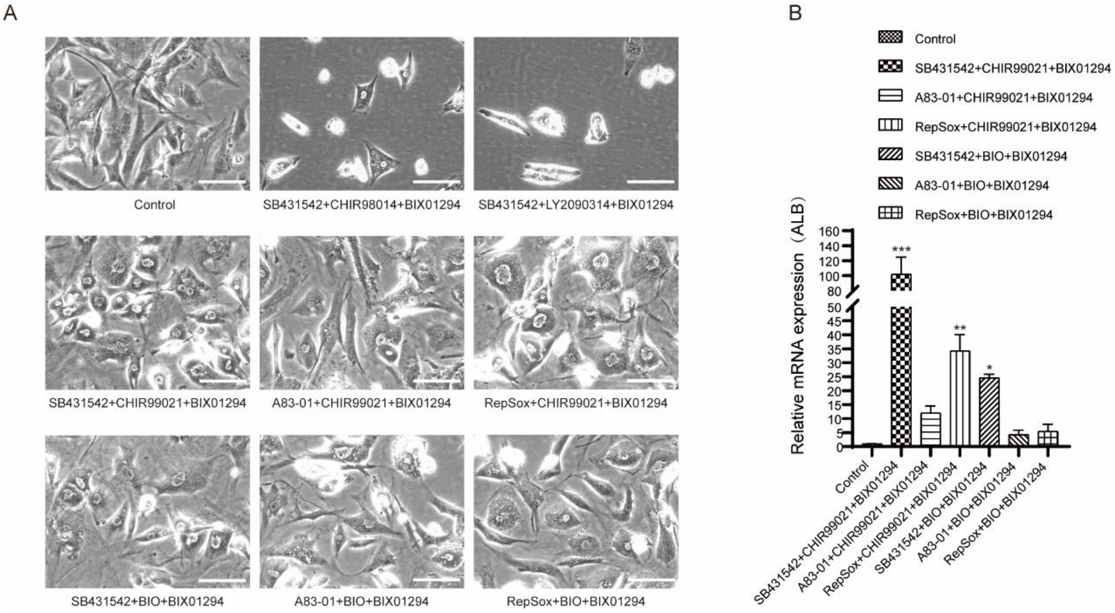

Supplementary Figure S2. Induction effect of different three-factor small molecule combinations. (**A**) Morphology of MEFs under treatment of different three-factor small molecule combinations. (**B**) RT-qPCR assay showing ALB expression of MEFs under treatment of different three-factor small molecule combinations. All scale bar, 100 μm.

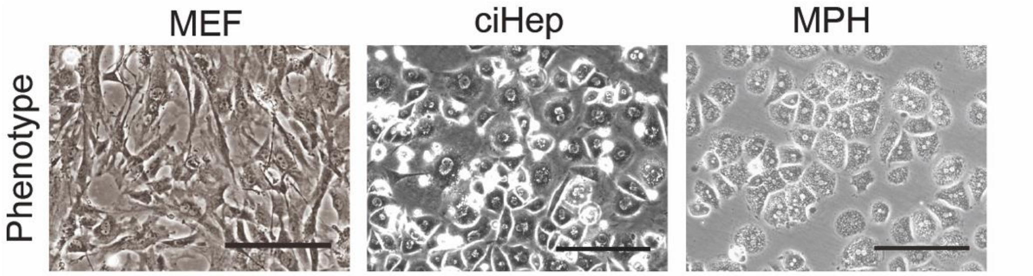

Supplementary Figure S3. Five-factor small molecule cocktail (SMC) for successful reprogramming of fibroblasts into hepatocyte-like cells. MEFs were homogeneously converted into ciHeps by the five-factor SMC including SB431543, CHIR99021, BIX01294, LDN193189 and DAPT.

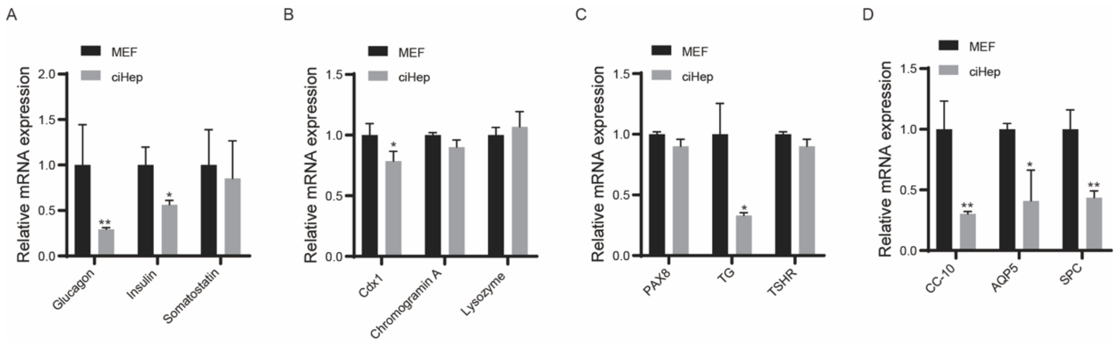

Supplementary Figure S4. Gene expression of other endoderm-derived cell types. **(A-D)** Marker gene expression of pancreatic cells (**A**), intestinal epithelia (**B**), thyroid epithelia (**C**) and lung alveolar epithelial (**D**) cells were detected by RT-qPCR.

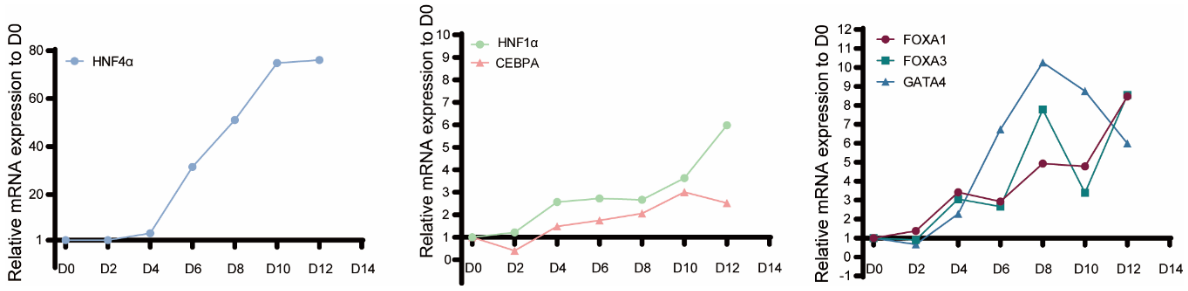

Supplementary Figure S5. TFs expression during MEFs reprogramming process. Hepatic lineage TFs enhanced expression during reprogramming process.

Supplementary Table

Supplementary Table S1. Small molecules information

**Full Name**	**Source**	**Molecule weight**	**Structure**	**Used concentration**
SB431542	Selleck, Cat. No. S1067	384.39	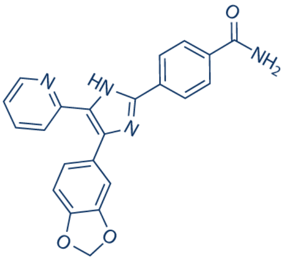	2-5 μM
CHIR99021	Selleck, Cat. No. S2924	501.8	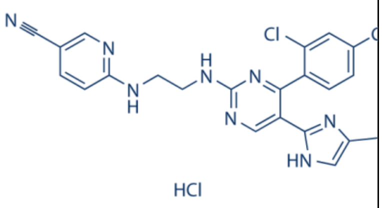	3-4 μM
LDN193189	Selleck, Cat. No. S2618	406.48	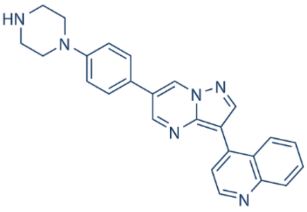	0.5 μM
BIX01294	Selleck, Cat. No. S8006	600.02	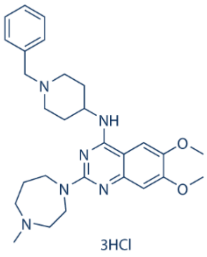	2 μM
DAPT	Selleck, Cat. No. S2215	432.46	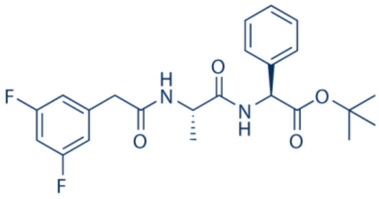	2 μM

Supplementary Table S2. Primer sequences

Primer Name	Sequence（5'-3'）
β-actin	F- GGCTGTATTCCCCTCCATCG
	R-CCAGTTGGTAACAATGCCATGT
HNF4α	F-AGACTCCACAGCCATCACCA
	R-AATGGCAGAGGGAGGCTTGA
HNF1α	F-GTGGCGAAGATGGTCAAGTC
	R-GCGTGGGTGAATTGCTGAG
ALB	F-TGTGTTGCCGATGAGTCTGC
	R-CGGAGGTTTGGAATGGCACA
CYP1A1	F-GCCGATCGGAGGTCTTTCTC
	R-AAGACCGCATCTGCACTTGG
CYP1A2	F-GACAATGGCGGTCTCATCCC
	R-TGCACGTTAGGCCATGTCAC
ACTA2	F-ACTACTGCCGAGCGTGAGAT
	R-CGTCAGGCAGTTCGTAGCTC
TIMP1	F-GAGACACACCAGAGCAGATACC
	R-GCTGGTATAAGGTGGTCTCGT
SNAI1	F-AGTTGACTACCGACCTTGCG
	R-GCTGGAAGGTGAACTCCACA
Vimentin	F-TTCTCTGGCACGTCTTGACC
	R-AGAGGTCAGCAAACTTGGACT
N-cadherin	F-AGCGCAGTCTTACCGAAGG
	R-TCGCTGCTTTCATACTGAACTTT
E-cadherin	F-GCCAGCGTCAACTGGACCAT
	R-TTGCAATCCTGCTGCCACGA
Sox2	F-GCGGAGTGGAAACTTTTGTCC
	R-CGGGAAGCGTGTACTTATCCTT
Nanog	F-TCTTCCTGGTCCCCACAGTTT
	R-GCAAGAATAGTTCTCGGGATGAA
AFP	F-CTTCCCTCATCCTCCTGCTAC
	R-ACAAACTGGGTAAAGGTGATGG
EpCAM	F-GCGGCTCAGAGAGACTGTG
	R-CCAAGCATTTAGACGCCAGTTT
Sox9	F-CCAGCAAGAACAAGCCACAC
	R-CTCTCGTTCAGCAGCCTCC
Oct4	F-GGAGGGATGGCATACTGTGG
	R-CTTTCATGTCCTGGGACTCCT
TBX3	F-AGATCCGGTTATCCCTGGGAC
	R-CAGCAGCCCCCACTAACTG
Sox17	F-GATGCGGGATACGCCAGTG
	R-CCACCACCTCGCCTTTCAC
CK18	F-GGAAGTGGATGCCCCCAAAT
	R-TGGTACTCTCCTCAATCTGCTG

CK19	F-GGGGGTTCAGTACGCATTGG
	R-GAGGACGAGGTCACGAAGC
DLK1	F-CCCAGGTGAGCTTCGAGTG
	R-GGAGAGGGGTACTCTTGTTGAG
LGR5	F-CCTACTCGAAGACTTACCCAGT
	R-GCATTGGGGTGAATGATAGCA
HNF6	F-GGCAACGTGAGCGGTAGTTT
	R-TTGCTGGGAGTTGTGAATGCT
HHEX	F-ATGGAGAAGACTGAAACAGGAGAA
	R-CGAACGATCCAAAGAGGCAC
c-Myc	F-GTTGGAAACCCCGCAGACA
	R-GTAGCGACCGCAACATAGGA
l-Myc	F-GGGAAACAATGCGCCTGC
	R-ATGTCCGCTCCCTCGCT
n-Myc	F-CCTTGAGCGACTCAGATGATGA
	R-GTCTTGGGACGCACAGTGAT
erbB2	F-GGCACTGTCTACAAGGGCAT
	R-GAGGCGGGACACATATGGAG
k-Ras	F-AGTAGACACGAAACAGGCTCA
	R-GCATCGTCAACACCCTGTCT
